# Bilateral atypical ovarian masses: don’t overlook a functional gonadotropic pituitary adenoma

**DOI:** 10.3389/fendo.2025.1597813

**Published:** 2025-06-23

**Authors:** Estelle Hagege, Geraldine Vitellius, Anne Fevre, Malorie Mostaert, Fabien Litre, Olivier Graesslin, Paul Pirtea, Brigitte Delemer

**Affiliations:** ^1^ Department of Endocrinology, Endocrinology and Gynecology, Reims University Hospital Center, Reims, France; ^2^ Department of Obstetrics, Gynecology and Reproductive Medicine, Reims University Hospital Center, Reims, France; ^3^ Department of Neurosurgery, Reims University Hospital Center, Reims, France; ^4^ Department of Obstetrics, Gynecology and Reproductive Medicine, Foch Hospital, Suresnes, France

**Keywords:** functional gonadotroph adenoma, pituitary adenoma, ovarian mass/cyst, ovarian hyperstimulation syndrome, follicle-stimulating hormone

## Abstract

Functional gonadotroph adenoma (FGA) is a rare condition associated with secretion of biologically active gonadotropins which affect reproductive organs. In women of reproductive age, it has been reported as a cause of spontaneous ovarian hyperstimulation syndrome (OHSS) occurring outside the context of assisted reproductive technology (ART). In rare instances, FGA may present as suspicious ovarian masses, leading to an overlooked pituitary disorder. We report the case of a 34-year-old woman initially suspected of having a bilateral ovarian tumor with a borderline component due to thick-walled cystic masses. She underwent pelvic surgery, resulting in an oophorectomy. However, a few weeks postoperatively, the sudden onset of galactorrhea prompted further investigation, revealing hyperprolactinemia, FSH hypersecretion, and low LH levels. Ultimately, the diagnosis of FGA was established. A literature review was conducted to analyze similar cases where patients underwent ovarian surgery without prior hormonal assessment or suspicion of pituitary pathology, only to be diagnosed with FGA later. Thirteen additional cases were identified, including ovarian cysts and two cases of suspicious ovarian masses, with diagnostic delays ranging from 1.5 to 10 years. This case highlights the importance of considering FGA in the differential diagnosis of bilateral ovarian masses to avoid unnecessary surgical procedures.

## 1 Introduction

Gonadotroph adenomas account for 15–40% of all pituitary adenomas and 80% of non-functioning adenomas (1, 2). As in most cases gonadotroph adenomas are non-functioning, their diagnosis is usually made through histopathological analysis. However, in rare instances, gonadotroph adenomas can be functional, most often secreting FSH (3). The clinical manifestations of functional gonadotroph adenomas (FGA) are varied and may arise either from tumor-related compression (4, 5) or, in women, from ovarian hyperstimulation syndrome (OHSS) (4–14) though the exact prevalence remains unclear. Other clinical presentations of FGA differ based on the patient’s sex and age, such as menstrual irregularities in women of reproductive age (4–10, 12–15), testicular enlargement in men (16–18), elevated testosterone levels (16, 17, 19) or precocious puberty in children (20–22).

The diverse and non-pathognomonic clinical features of FGA often lead to significant diagnostic delays, sometimes up to 15 years from the onset of symptoms to diagnosis (1). Furthermore, in a few cases, attention to hyperstimulated ovaries has led to erroneous diagnoses, sometimes resulting in inappropriate surgical procedures.

Herein we report the case of a 34-year-old patient with bilateral multicystic ovaries, initially suspected to have bilateral mucinous cystadenomas with concern about a borderline component, leading to a bilateral oophorectomy. A few weeks after the surgery, a functional pituitary macroadenoma was discovered and surgically confirmed. We then carried out a literature review to identify similar cases and to describe ovarian pathologies that had been mistakenly evoked in these patients.

## 2 Case presentation

This is a 34-year-old woman, mother of two children, who presents to the gynecological emergency department with acute pelvic pain. Her spontaneous, non-contraceptive cycles are prolonged, characterized by spaniomenorrhea. She reported no associated headaches, visual disturbances, or other neuro-ophthalmologic symptoms at the time of presentation.

**Figure 1:**
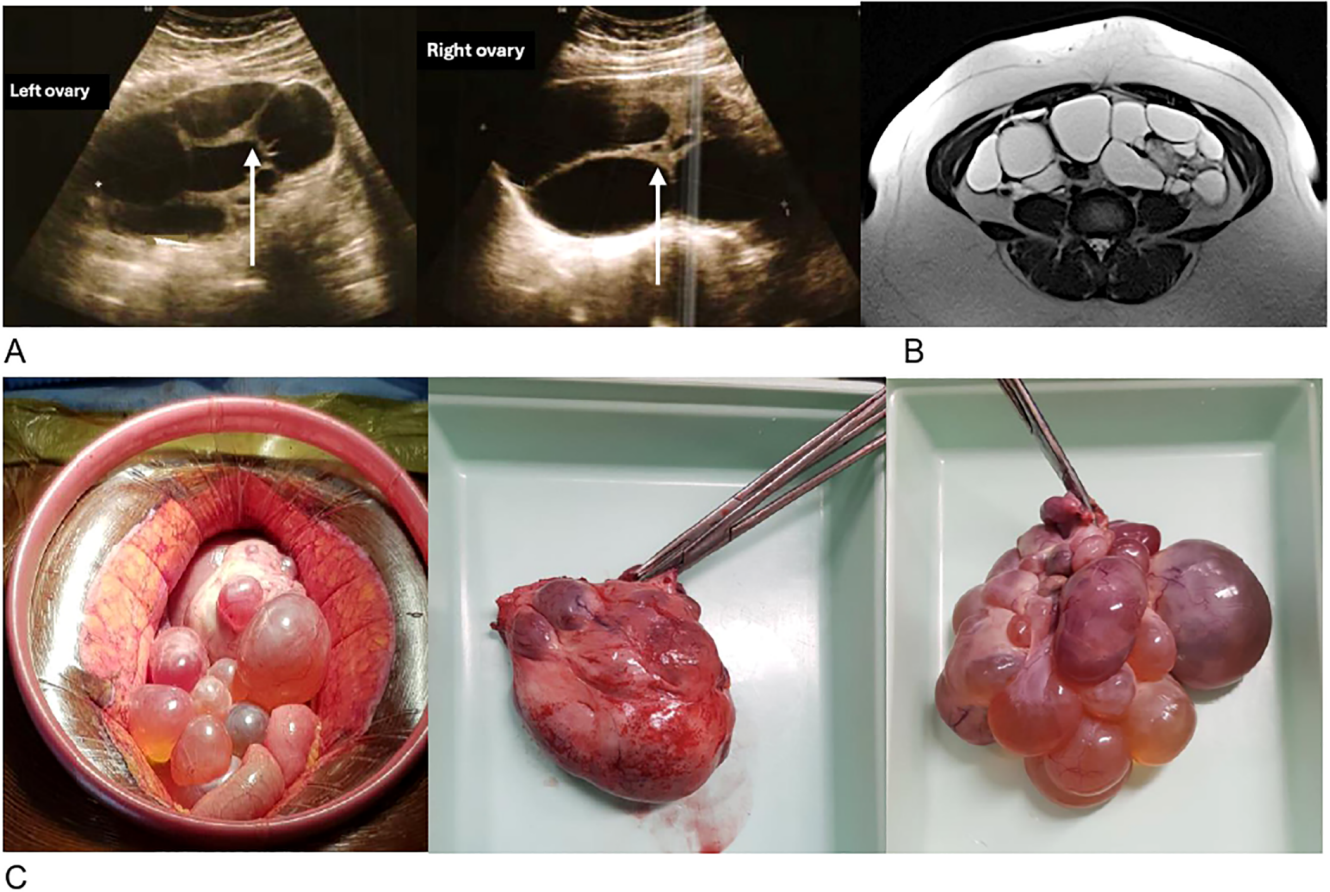
Morphological radiological and surgical appearance of the ovaries. **(A)** Transvaginal ultrasound of multicystic ovaries with thick walls (arrow). **(B)** Axial section of the pelvic MRI in T2 showing bilateral ovarian cysts. **(C)** Pre- and post-operative photographs of the left ovary weighing 268g (left) and the right ovary weighing 390g (right).

The pelvic ultrasound shows multicystic ovaries, right (143x79mm) and left (117x71mm), with thick septa and no hypervascularization on Doppler (**Figure 1A**). There is no pelvic effusion. The uterus is globular with an adenomyomatous appearance and a posterior fibroid. The endometrium is regular, measuring 6.3 mm. The pelvic MRI (**Figure 1B**) describes multilocular bilateral ovarian cysts extending 4 cm on the left and 12 cm on the right, with irregular septa and tissue portions accompanied by strong diffusion hypersignal without restriction of the apparent diffusion coefficient (ADC). The diagnosis of bilateral mucinous cystadenoma is considered, with suspicion of a borderline component within the thick walls. The tumor markers show an elevated Ca 125 antigen at 42.4 U/ml (N <35), a normal ACE marker at 1.1 ng/ml (N <3), and a normal CA 19.9 antigen at 19.4 U/ml (N <35).

After a national multidisciplinary discussion of rare gynecological tumors, a bilateral surgical intervention was decided, with cystectomy when possible, knowing that a bilateral adnexectomy could be the only technical option.

The patient is operated on by laparoscopy with conversion to laparotomy. As cystectomy proves impossible, a bilateral adnexectomy with omentectomy is performed (**Figure 1C**).

The pathological examination concludes with large bilateral ovarian fibromas associated with numerous functional cysts, without histological signs of malignancy.

A few weeks after surgery, spontaneous galactorrhea appears, revealing hyperprolactinemia at 76 ng/ml (N: 2.74-19.7), rechecked at 85.9 ng/ml. Estradiol is 28 pg/ml under hormone replacement therapy, FSH is elevated but moderately in this ovariectomized woman at 20.5 UI/L (N menopause >25), and LH is very low at 1 UI/L. The corticotropic, thyrotropic, and somatotropic axes show no insufficiency or hypersecretion.

**Figure 2:**
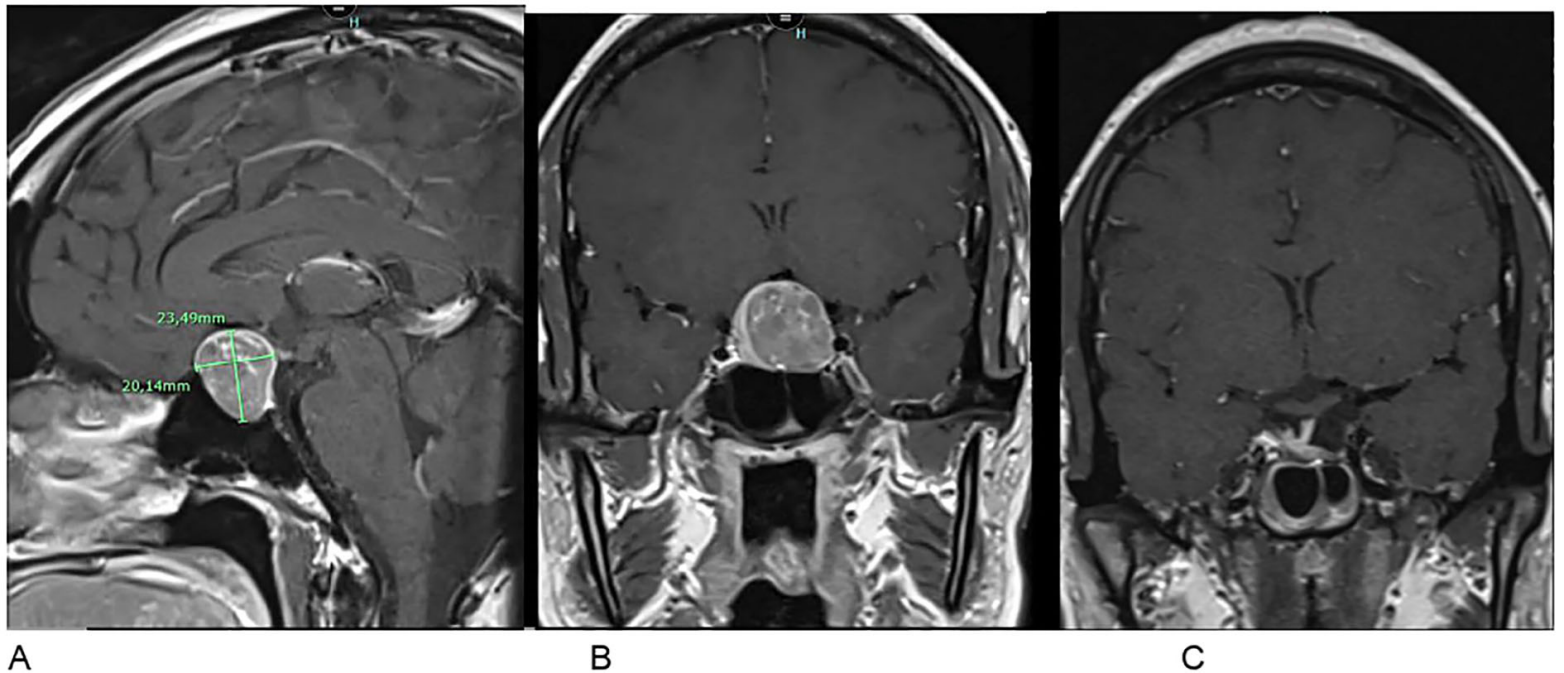
Pituitary MRI in T1-weighted post-contrast sequences. **(A, B)** Preoperative frontal and sagittal views showing a pituitary macroadenoma (23.5 × 20.1 mm) with optic chiasm involvement. **(C)** Postoperative frontal view at 3 months showing regression of the tumor mass.

The pituitary MRI reveals a macroadenoma measuring 23.5 x 20.1 mm compressing the optic chiasm without invading the left cavernous sinus (**Figures 2A, B**).

A surgical approach was then undertaken. Histopathological analysis, including hormonal immunohistochemistry, revealed a gonadotroph macroadenoma with intense and diffuse FSH expression and focal LH staining. The Ki-67 proliferation index was estimated at a maximum of 2%, and p53 exhibited a wild-type staining pattern. At three months postoperatively, hormonal assessment showed a decrease in prolactin to 0.6 ng/mL and an increase in LH to 8.6 IU/L. FSH levels remained elevated at 19.6 IU/L, which is consistent with the patient’s ovariectomized status. The remaining pituitary axes were within normal limits. Postoperative MRI at three months demonstrated a small residual lesion in the sellar region, requiring continued radiological surveillance (**Figure 2C**).

## 3 Review of the literature (1985-2024)

We performed a systematic review covering the period from 1985 to 2024. A literature search was conducted using the following keyword combinations: “pituitary adenoma” and “ovarian mass” or “ovarian hyperstimulation” through PubMed. Reviews and commentaries were excluded.

**Figure 3:**
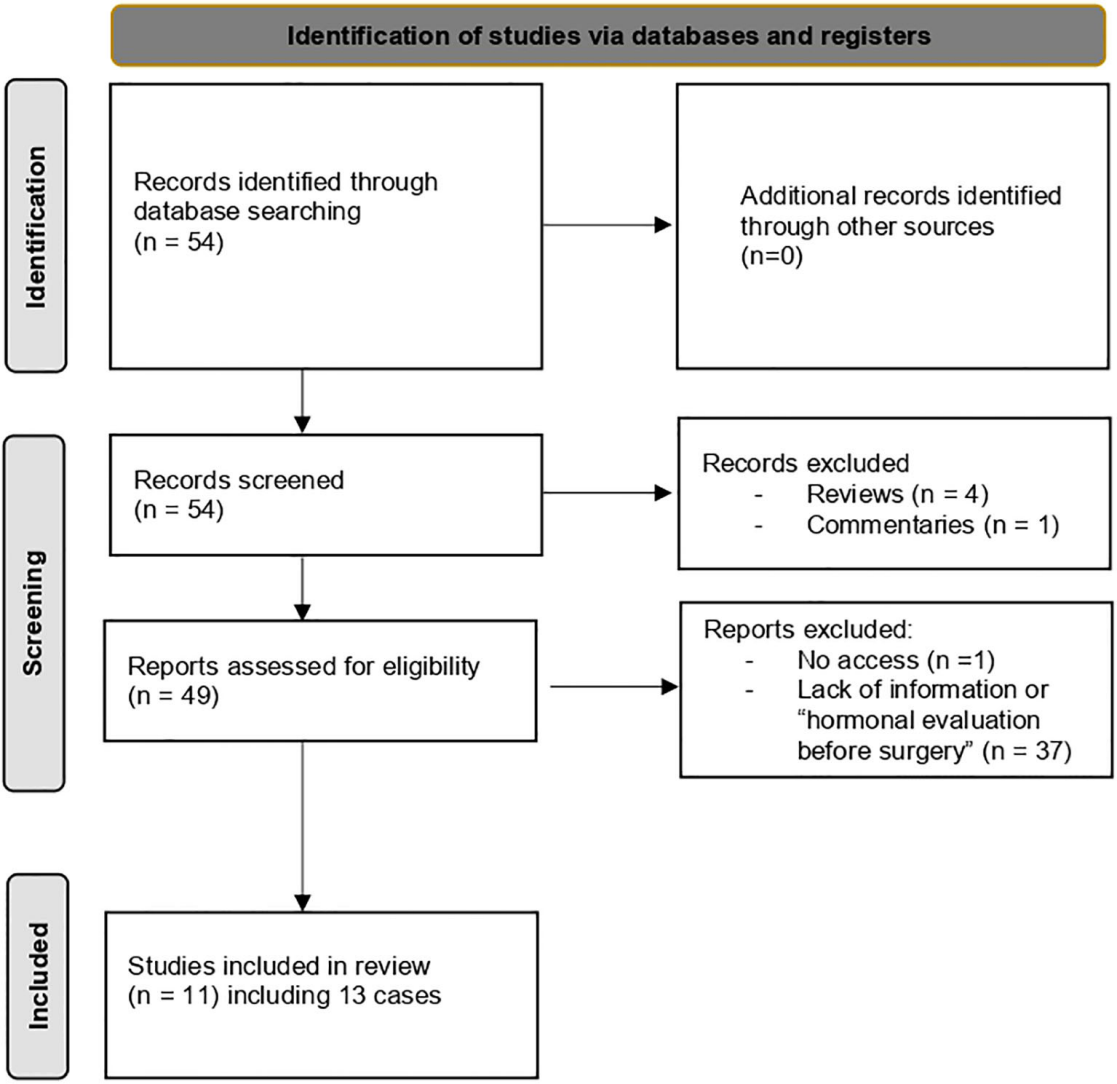
Diagnostic flowchart based on literature review (1985–2024): ovarian surgery prior to FGA diagnosis.

A total of 54 articles of FGA with ovarian involvement was identified. Among these, we selected cases where patients underwent ovarian surgery prior to a hormonal evaluation of the gonadotropic axis, leading to a delayed diagnosis of FGA like our case. To our knowledge, 13 such cases have been reported (**Figure 3**). Clinical features, pathological results, and therapeutic interventions were extracted from each study and systematically summarized (**Table 1**).

**Table 1:** Clinical features of women who underwent ovarian surgery before diagnosis of a functioning gonadotroph adenoma.

	Case number	Age (years)	Presenting compliant:	Menstrual irregulariti es	Ultrasound Findings	Ovarian Surgery	Anapath ovarian surgery	Delay between first visit/ adenoma diagnostic	Pituitary adenoma size (mm)	Pre- operative*	Post- operative*	Immunocyto chemistry
Our case	Case 1	34	Abdominal pain	Yes	Bilateral cystic ovaries with thick septa. Suspicion of borderline tumor.	Bilateral oophorectomy	Follicular cysts	1.5 years	23.5	FSH: 20.5 LH: 1 E2: 28	FSH: 19.4 LH: 8.6	Positive for FSH, LH focal
(1)	Case 2	40	Menometrorra gia and Galactorrhea (15 years)	Yes	OHSS	Oophorocyste ctomy (3 times)	Luteinized follicular cysts	X	27	FSH 19.2 LH 0.6 E2 1 041	X	Positive for FSH and LH
(2)	Case 3	36	Abdominal pain and metrorrhagia	X	Bilateral cystic ovaries	Oophorocyste ctomy	Follicular cysts	3 years	18	FSH: 10.9 LH < 0.1 E2: 304	FSH: 8.8 LH: 3.1 E2: 99	Positive for FSH, negative for LH
(7)	Case 4	35	Abdominal pain	X	Ovarian torsion secondary to bilateral enlarged polycystic ovaries	Right salpingo- oophorectomy	Follicular cyst	X	34	FSH: 17.3 LH <0.1 E2 130	FSH: 8.3 LH: 2.1 E2: 89	Positive for FSH
(14)	Case 5	34	Infertility	Yes	Right cystic ovary.	Right oophorectomy	X	5 years	54	FSH: 38.7 LH: 36 E2: 3740	FSH: 10.5 LH: 6.8 E2: 72	Positive for FSH and LH
(23)	Case 6	40	Metrorrhagia	Yes	Bilateral cystic ovaries with ascites. Suspicion of malignant tumor.	Bilateral oophorocystec tomy	Follicular cysts	X	X	FSH: 10.7 LH < 0.1 E2: 127	X	Positive for FSH, negative for LH
(24)	Case 7	43	Abdominal pain	Yes	Bilateral cystic ovaries. Suspicion of granulosa cell tumor.	Hysterectomy with bilateral salpingooopho rectomy	Follicular cysts	X	61	FSH: 56 LH: 1.1 E2: 62	X	X
(25)	Case 8	36	Abdominal pain	X	Bilateral ovarian cysts (right: 40 mm, left: 52 mm)	Bilateral oophorectom y	Follicular cysts	1.5 years	15	FSH 8.7 LH 0.8 E2 571	FSH 3.2 LH 1.8 E2 < 27	Positive for FSH
(39)	Case 9	32	Abdominal pain	No	Irregular mass of multiple cysts (168 x 146 x 94 mm).	Oophorocyst ectomy (3 times)	Follicular cysts	10 years	Large macroad enoma	FSH: 26.7 LH < 0.5 E2: 1562	FSH: 14 LH: 0.5 E2: 218	X
(39)	Case 10	27	Abdominal pain	Yes	Large semisolid/cysti c mass (140 mm).	Oophorocyst ectomy	Follicular cysts	5 years	48	FSH: 32 LH < 0.2 E2: 967	FSH: 6.8 LH: 4.5 E2: 13	Positive for FSH, and occasional LH and TSH cells
(39)	Case 11	25	Abdominal pain	Yes	Right ovarian torsion with cysts and cystic left ovarian.	Right salpingo- oophorectom y	X	1.5 years	Large pituitary tumor	FSH: 10.4 LH < 0.5 E2: 262	FSH: 5.5 LH: 2	Positive for FSH and LH
(40)	Case 12	29	Oligomeno rrhea	X	Bilateral ovarian cysts, and bilateral adnexal torsion	Right oophorectom y	X	X	25	FSH 6.8 LH 0.1 E2 237	FSH 5.9 LH 2.5 E2 144	Positive for FSH
(36)	Case 13	23	Oligomeno rrhea	Yes	Bilateral cystic ovaries	Bilateral oophorocyste ctomy	Follicular and luteinized follicular epithelium	2 years	28	FSH: 13.4 LH: 0.5 E2: 1675	FSH: 4.03 LH: 3.48 E2: 79	Positive for b- HCG, LH, and only <5% for FSH
(52)	Case 14	29	Abdominal pain	Yes	Bilateral cystic ovaries (up to 200 mm)	Hysterectom y with bilateral salpingoooph orectomy	Follicular cysts	2, 5 years	23	FSH: 6.8 LH: 0.1 E2: 237	X	Positive for FSH, negative for LH

*FSH, follicle stimulating hormone (IU/L); LH, luteinizing Hormone (IU/L); E2, Estradiol (pg/ml).*

All patients initially presented to gynecology departments. The primary symptoms prompting consultation included abdominal pain, metrorrhagia, menstrual cycle irregularities, and infertility. Pelvic ultrasound findings revealed ovarian enlargement, ovarian cysts, ovarian OHSS, or, in three cases including our patient (23, 24), suspected malignant ovarian masses. One case involving ovarian surgery and recurrences showed rectal involvement with fibromatosis, raising concerns about malignancy (25).

The surgical procedures performed ranged from cystectomy to unilateral or bilateral oophorectomy with or without salpingectomy. In one case, a hysterectomy was performed (24), and in another, a rectotomy was required (25). Histopathological findings consistently indicated benign ovarian masses. The rectotomy case revealed fibromatosis (desmoid tumor) with no evidence of malignancy.

In our case, after bilateral oophorectomy, the FSH level was elevated at 20.5 IU/L, but the LH level remained low at 1 IU/L. In 13 on 14 cases in our review of literature (**Table 1**), hormonal analyses also showed normal or elevated FSH levels with low LH levels and an increased FSH/LH ratio. In cases where ovarian tissue was preserved, estradiol was increased (like situations of ovarian hyperstimulation).

The time interval between the initial consultation and the diagnosis of pituitary adenoma ranged from 1.5 to 10 years. The diagnosis of FGA was established based on recurrent ovarian abnormalities (6 out of 14 cases), galactorrhea (3 out of 14 cases) as seen in our patient—one of whom had a 15-year history of galactorrhea without an established diagnosis—or visual disturbances and/or headaches (3 out of 14 s). In one case, the pituitary adenoma was identified during an investigation for amenorrhea. After pituitary surgery, hyperstimulation was controlled, and LH secretion normalized in 9 out of 10 cases with available postoperative assessments.

## 4 Discussion

FGA cases remain rare and poorly recognized, despite a growing number of reports in the literature documenting occurrences in both men and women (7, 26, 27). FGAs have been associated with various clinical manifestations, including menstrual irregularities (oligomenorrhea, spaniomenorrhea, menorrhagia, amenorrhea) (4–10, 12–15), abdominal pain (4–7, 9, 11–14), tumor-related symptoms such as headaches and visual disturbances (4, 5), as well as OHSS in women of reproductive age (4–14).

However, the presentation of suspected ovarian masses—later identified as a consequence of FGA—can be particularly challenging, potentially leading to diagnostic errors and significant delays in treatment. We report the case of a patient with bilateral ovarian masses, initially raising suspicion of an ovarian tumor with a borderline component due to the presence of thickened septations separating the atypical cysts. The diagnostic challenge in our case led to an oophorectomy before ultimately discovering a FGA. In our literature review, 13 other cases were identified with similar clinical presentations, including ovarian involvement without prior hormonal assessment, leading to diagnostic delays.

Malignant ovarian tumors were suspected in three cases from our literature review, despite their usual occurrence in older patients (4) and their predominantly unilateral presentation. However, serous borderline ovarian tumors, as suspected in our case, can be bilateral in 15%-25% of cases, and noninvasive peritoneal spread (as observed in case 6 and 7) can be seen in 15%-40% of cases (28). Notably, two of the five IOTA classification criteria for malignant tumors (29) could have been observed in these patients, such as the presence of ascites (case 6) in a context of hyperestrogenism and the presence of irregular septations with the largest diameter exceeding 100 mm. Furthermore, in the WHO classification of ovarian tumors (32, 33), FGA is not mentioned as a differential diagnosis, likely due to the rarity of this diagnosis. Additionally, hormonal evaluation is not systematically recommended.

Ovarian involvement in FGA must also be differentiated from polycystic ovary syndrome (PCOS). In PCOS, clinical signs of hyperandrogenism are present and follicles rarely exceed 10 mm in size. However, hormonal evaluation reveals a completely different profile, with elevated LH and testosterone levels (4, 30). OHSS was also a differential diagnosis, but it is most often iatrogenic, occurring after the administration of gonadotropins in the context of Assisted Reproductive Technology (ART). Functional ovarian cysts associated with FGA can sometimes trigger OHSS and, in rare cases, progress to a severe form (23). In other rare cases of spontaneous OHSS, activating mutations in the FSH receptors have been identified (31, 32). Others of unilateral or bilateral ovarian cysts can also be observed in patients with McCune-Albright syndrome due to a somatic mutation in the GNAS gene (guanine nucleotide-binding protein, alpha stimulating), leading to ovarian autonomy and hyperestrogenism, with low FSH levels (33).

The FSH–LH dissociation observed in FGA is not fully understood, but several hypotheses have been proposed. First, elevated estradiol levels may exert negative feedback on LH secretion, while sparing the autonomous secretion of FSH by the tumor (10, 34). A similar hormonal profile—elevated FSH with suppressed LH—has also been described in the context of ectopic FSH secretion by a pancreatic neuroendocrine tumor (35). Compression of normal pituitary tissue by the adenoma may further contribute to impaired LH secretion (36, 37). Interestingly, experimental and clinical data support the idea that FSH itself may directly suppress LH secretion, independently of estradiol levels (23, 38). This mechanism is further supported by our patient’s hormonal profile after bilateral oophorectomy, where estradiol levels were low, but LH remained suppressed. Overall, this dissociation profile (suppressed LH) is consistently observed across nearly all reported cases, regardless of tumor size, and even in cases of ectopic hormone secretion.

In all cases of FGA-related OHSS or ovarian mass requiring surgery that were documented from the literature, no significant pathological abnormalities were found in the ovaries.

However, in the cases we reviewed, some surgeries seemed unavoidable due to acute complications, such as adnexal torsion that happened in 3 cases (7, 39, 40) or due to recurrent ovarian cysts (14, 26). In our literature review, 43% of ovarian surgeries resulted in unilateral or bilateral oophorectomy, as was the case for our patient, since cystectomy was not technically feasible.

Unfortunately, these surgeries, although frequently extended to the whole ovary, did not led to a correct diagnosis, as the diagnosis of pituitary adenoma was delayed by several years after ovarian surgery, with recurrence of symptoms and cysts. The necessity of urgent pituitary treatment has been well reported in the series described by Graillon et al. (12). Indeed, pituitary resection of the adenoma can reduce ovarian size within days after surgery and lead to complete remission of OHSS. In our review, 9 out of 10 cases with available postoperative assessments showed normalization of the hormonal profile, including control of hyperestrogenism, normalization of LH levels, and a decrease in the FSH/LH ratio. However, some cases of adnexal torsion have been described even after successful pituitary surgery and in some cases, control of the pituitary adenoma is not possible with surgery alone.

From a therapeutic perspective, several medications have been administered to patients with FGA and appear to be beneficial. Dopamine agonists (bromocriptine, cabergoline) are effective in reducing ovarian size, leading to decreased estradiol levels in treated patients (41, 42). Their anti-VEGF (vascular endothelial growth factor) effect helps prevent OHSS by inhibiting VEGF, thereby reducing vascular permeability and ovarian swelling. This makes these treatments useful in preventing OHSS during ART (43, 44), although their direct impact on FSH secretion remains unproven. However, these treatments do not appear to significantly decrease the size of the pituitary adenoma (4, 5, 42). Interestingly, a case reported by Ceccato et al. (19) showed a modest reduction in macroadenoma size—from 68×64×60 mm to 59×46×60 mm—following a combined treatment with octreotide and cabergoline. However, surgical intervention was still required six months later. Similarly, somatostatin analogs like octreotide (4, 36, 45) have shown no effect on adenoma size. Treatment outcomes with GnRH analogs appear to vary depending on the specific class used. While GnRH agonists have been associated with a potential increase in tumor volume due to their initial stimulatory “flare-up” effect (46, 47), GnRH antagonists may offer a more favorable profile. In one reported case (48), the combination of a GnRH antagonist (Ganirelix) with cabergoline resulted in a marked drop in estradiol levels within a month and a noticeable reduction in ovarian size. This outcome may be linked not only to the suppression of gonadotropin secretion at the pituitary level but also to a possible direct inhibitory effect on granulosa cell steroidogenesis, particularly aromatase activity, contributing to reduced estrogen synthesis and symptomatic relief (49). Despite the potential benefits of these medical therapies, only pituitary surgery has consistently resulted in symptom regression and normalization of hormone levels (1, 42, 50). Moreover, several cases of spontaneous pregnancy following surgical resection have been documented, reinforcing the role of surgery as a definitive therapeutic approach (46, 47). Nevertheless, long-term follow-up is necessary due to the risk of recurrence of these large pituitary adenomas (5, 10, 15).

**Figure 4:**
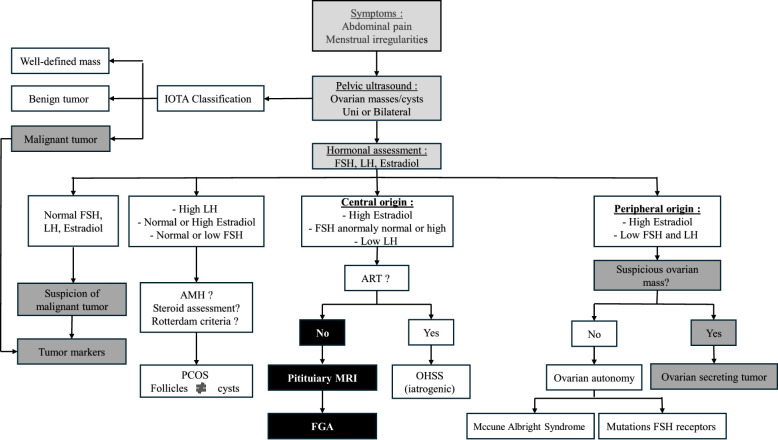
Diagnostic algorithm for ovarian masses and cysts: role of hormonal and imaging evaluation.

Finally, cases of patients with abdominal pain, menstrual cycle disorders, unilateral or bilateral ovarian cysts, or suspicious ovarian masses should prompt a hormonal assessment of sex hormones upfront to avoid missing a central cause of hyperestradiolism (51) and potentially prevent unnecessary pelvic surgery. Ultimately, ovarian cysts associated with low LH levels or unsuppressed FSH with high estradiol should raise suspicion for FGA and warrant further investigation (**Figure 4**).

Nevertheless, several limitations should be acknowledged. First, this case report is limited by its single-patient nature. Furthermore, the retrospective literature review may have missed unpublished or unindexed cases. In addition, the lack of long-term postoperative follow-up data in some cases limits the assessment of recurrence risk and long-term endocrine outcomes. Finally, the absence of a centralized clinical registry restricts our understanding of the true prevalence and the full clinical spectrum of FGA.

## 5 Conclusion

We described the case of a patient with bilateral ovarian cysts with suspicious septations, which led to bilateral oophorectomy before the later discovery of a functional FSH-secreting gonadotroph adenoma. To our knowledge, thirteen similar cases, where ovarian surgery preceded hormonal evaluation, have been reported, along with numerous cases of OHSS due to FGA. A prior hormonal evaluation might have prevented such surgeries in these young women, but the rarity of this diagnosis and limited medical awareness continue to pose diagnostic challenges. Pituitary surgery remains the first-line treatment and requires long-term monitoring of both pituitary and ovarian function. Given the diagnostic complexity and potential reproductive consequences, multicenter studies and the creation of a clinical registry are needed to improve early recognition, standardize management, and reduce unnecessary interventions.

## References

[1] Cooper O Geller JL Melmed S. Ovarian hyperstimulation syndrome caused by an FSH-secreting pituitary adenoma. Nat Clin Pract Endocrinol Metab. (2008) 4:234−8. doi: 10.1038/ncpendmet0758 18268519 PMC2777809

[2] Kawaguchi T Ogawa Y Ito K Watanabe M Tominaga T. Follicle-stimulating hormone-secreting pituitary adenoma manifesting as recurrent ovarian cysts in a young woman–latent risk of unidentified ovarian hyperstimulation: a case report. BMC Res Notes. (2013) 6:408. doi: 10.1186/1756-0500-6-408 24119690 PMC3852055

[3] Mayson SE Snyder PJ. Silent (clinically nonfunctioning) pituitary adenomas. J Neurooncol. (2014) 117:429−36. doi: 10.1007/s11060-014-1425-2 24676675

[4] Ntali G Capatina C Grossman A Karavitaki N. Clinical review: Functioning gonadotroph adenomas. J Clin Endocrinol Metab. (2014) 99:4423−33. doi: 10.1210/jc.2014-2362 25166722

[5] Halupczok J Kluba-Szyszka A Bidzińska-Speichert B Knychalski B. Ovarian hyperstimulation caused by gonadotroph pituitary adenoma–review. Adv Clin Exp Med. (2015) 24:695−703. doi: 10.17219/acem/25212 26469116

[6] Caretto A Lanzi R Piani C Molgora M Mortini P Losa M. Ovarian hyperstimulation syndrome due to follicle-stimulating hormone-secreting pituitary adenomas. Pituitary. (2017) 20:553−60. doi: 10.1007/s11102-017-0817-7 28676954

[7] Cote DJ Smith TR Sandler CN Gupta T Bale TA Bi WL. Functional gonadotroph adenomas: case series and report of literature. Neurosurgery. (2016) 79:823−31. doi: 10.1227/NEU.0000000000001188 26692108 PMC4912468

[8] Castelo-Branco C del Pino M Valladares E. Ovarian hyperstimulation, hyperprolactinaemia and LH gonadotroph adenoma. Reprod BioMed Online. (2009) 19:153−5. doi: 10.1016/S1472-6483(10)60065-X 19712547

[9] Wada-Hiraike O Yamada S Osuga Y. An extremely rare case of pituitary functioning gonadotroph microadenoma accompanied by ovarian hyperstimulation syndrome in a woman of reproductive age. F S Rep. (2022) 3:79−83. doi: 10.1016/j.xfre.2022.01.006 35386509 PMC8978087

[10] Djerassi A Coutifaris C West VA Asa SL Kapoor SC Pavlou SN. Gonadotroph adenoma in a premenopausal woman secreting follicle-stimulating hormone and causing ovarian hyperstimulation. J Clin Endocrinol Metab. (1995) 80:591−4. doi: 10.1210/jcem.80.2.7852525 7852525

[11] Davis JRE McNeilly JR Norris AJ Pope C Wilding M McDowell G. Fetal gonadotrope cell origin of FSH-secreting pituitary adenoma - insight into human pituitary tumour pathogenesis. Clin Endocrinol (Oxf). (2006) 65:648−54. doi: 10.1111/j.1365-2265.2006.02644.x 17054468

[12] Graillon T Castinetti F Chabert-Orsini V Morange I Cuny T Albarel F. Functioning gonadotroph adenoma with severe ovarian hyperstimulation syndrome: A new emergency in pituitary adenoma surgery? Surgical considerations and literature review. Ann Endocrinol (Paris). (2019) 80:122−7. doi: 10.1016/j.ando.2018.11.007 30825998

[13] Eisenberg A Mersereau J Buckley AF Gratian L. Multiple pituitary adenomas with functional follicle-stimulating hormone secretion leading to ovarian hyperstimulation syndrome. AACE Clin Case Rep. (2019) 5:e159−63. doi: 10.4158/ACCR-2018-0474 31967024 PMC6873869

[14] Hasegawa H Nesvick CL Erickson D Cohen SC Yolcu YU Khan Z. Gonadotroph pituitary adenoma causing treatable infertility and ovarian hyperstimulation syndrome in female patients: neurosurgical, endocrinologic, gynecologic, and reproductive outcomes. World Neurosurg. (2021) 150:e162−75. doi: 10.1016/j.wneu.2021.02.115 33684575

[15] Young WF Scheithauer BW Kovacs KT Horvath E Davis DH Randall RV. Gonadotroph adenoma of the pituitary gland: a clinicopathologic analysis of 100 cases. Mayo Clin Proc. (1996) 71:649−56. doi: 10.1016/S0025-6196(11)63002-4 8656706

[16] Pigny P Henric B Lahlou N Christin S Mazzuca M Dewailly D. A gonadotroph adenoma with a high proportion of basic FSH isohormones by chromatofocusing. J Clin Endocrinol Metab. (1996) 81:2407–8. doi: 10.1210/jcem.81.6.8964889 8964889

[17] Chamoun R Layfield L Couldwell WT. Gonadotroph adenoma with secondary hypersecretion of testosterone. World Neurosurg. (2013) 80:900.e7–11. doi: 10.1016/j.wneu.2012.11.069 23201183

[18] Dahlqvist P Koskinen LOD Brännström T Hägg E. Testicular enlargement in a patient with a FSH-secreting pituitary adenoma. Endocrine. (2010) 37:289−93. doi: 10.1007/s12020-009-9302-z 20960265

[19] Ceccato F Occhi G Regazzo D Randi ML Cecchin D Gardiman MP. Gonadotropin secreting pituitary adenoma associated with erythrocytosis: case report and literature review. Hormones (Athens). (2014) 13:131−9. doi: 10.1007/BF03401328 24722134

[20] Vargas G Balcazar-Hernandez LJ Melgar V Magriña-Mercado RM Gonzalez B Baquera J. An FSH and TSH pituitary adenoma, presenting with precocious puberty and central hyperthyroidism. Endocrinol Diabetes Metab Case Rep. (2017) 2017:17−0057. doi: 10.1530/EDM-17-0057 PMC5510394 28721217

[21] Clemente M Caracseghi F Gussinyer M Yeste D Albisu M Vázquez E. Macroorchidism and panhypopituitarism: two different forms of presentation of FSH-secreting pituitary adenomas in adolescence. Horm Res Paediatr. (2011) 75:225−30. doi: 10.1159/000322211 21196695

[22] Ceraudo M Criminelli Rossi D Di Iorgi N Cama A Piatelli G Consales A. Pediatric pituitary adenoma with mixed FSH and TSH immunostaining and FSH hypersecretion in a 6 year-old girl with precocious puberty: case report and multidisciplinary management. Int J Neurosci. (2022) 132:362−9. doi: 10.1080/00207454.2020.1815734 32842843

[23] Kanaya M Baba T Kitajima Y Ikeda K Shimizu A Morishita M. Continuous follicle-stimulating hormone exposure from pituitary adenoma causes periodic follicle recruitment and atresia, which mimics ovarian hyperstimulation syndrome. Int J Womens Health. (2012) 4:427−31. doi: 10.2147/IJWH.S33386 23071411 PMC3469228

[24] Mor E Rodi IA Bayrak A Paulson RJ Sokol RZ. Diagnosis of pituitary gonadotroph adenomas in reproductive-aged women. Fertil Steril. (2005) 84:757. doi: 10.1016/j.fertnstert.2005.02.050 16169418

[25] Broughton C Mears J Williams A Lonnen K. A clinically functioning gonadotroph adenoma presenting with abdominal pain, ovarian hyperstimulation and fibromatosis. Endocrinol Diabetes Metab Case Rep. (2018). https://edm.bioscientifica.com/view/journals/edm/2018/1/EDM18-0123.xml. 10.1530/EDM-18-0123 PMC6300858 30532999

[26] Wang L Liang H Deng C Yu Q Gong F Feng F. Functioning gonadotroph adenomas in premenopausal women: clinical and molecular characterization and review of the literature. Pituitary. (2022) 25:454−67. doi: 10.1007/s11102-021-01205-9 35138520

[27] He Y Gao YT Sun L. Functioning gonadotroph adenoma with hyperestrogenemia and ovarian hyperstimulation in a reproductive-aged woman: A case report and review of literature. World J Clin cases. (2023) 11:1341−8. doi: 10.12998/wjcc.v11.i6.1341 36926127 PMC10013118

[28] Vancraeynest E Moerman P Leunen K Amant F Neven P Vergote I. Fertility preservation is safe for serous borderline ovarian tumors. Int J Gynecol Cancer. (2016) 26:1399−406. doi: 10.1097/IGC.0000000000000782 27465897

[29] Timmerman D Ameye L Fischerova D Epstein E Melis GB Guerriero S. Simple ultrasound rules to distinguish between benign and Malignant adnexal masses before surgery: prospective validation by IOTA group. BMJ. (2010) 341:c6839. doi: 10.1136/bmj.c6839 21156740 PMC3001703

[30] Mayur O Elshimy G Bansal R Jacob A Raj R. A case of undiagnosed functional gonadotroph adenoma leading to ovarian hyperstimulation syndrome. Cureus. (2022). https://www.cureus.com/articles/101699-a-case-of-undiagnosed-functional-gonadotroph-adenoma-leading-to-ovarian-hyperstimulation-syndrome. 10.7759/cureus.26242 PMC9312281 35911295

[31] Binder H Dittrich R Hager I Muller A Oeser S Beckmann MW. Association of FSH receptor and CYP19A1 gene variations with sterility and ovarian hyperstimulation syndrome. Reproduction. (2008) 135:107−16. doi: 10.1530/REP-07-0276 18159088

[32] Smits G Olatunbosun O Delbaere A Pierson R Vassart G Costagliola S. Ovarian hyperstimulation syndrome due to a mutation in the follicle-stimulating hormone receptor. N Engl J Med. (2003) 349:760−6. doi: 10.1056/NEJMoa030064 12930928

[33] Pirtea P Heggarty E Hagege E Tran C De Ziegler D Farabet C. Successful ART outcome in a woman with McCune-Albright syndrome: a case report and literature review. J Assist Reprod Genet. (2023) 40:1669−75. doi: 10.1007/s10815-023-02844-6 37278881 PMC10352189

[34] Välimäki MJ Tiitinen A Alfthan H Paetau A Poranen A Sane T. Ovarian hyperstimulation caused by gonadotroph adenoma secreting follicle-stimulating hormone in 28-year-old woman. J Clin Endocrinol Metab. (1999) 84:4204−8. doi: 10.1210/jcem.84.11.6138 10566673

[35] Miras AD Mogford JT Wright J Mendoza NN Xekouki P Lakhani A. Ovarian hyperstimulation from ectopic hypersecretion of follicle stimulating hormone. Lancet. (2015) 385:392. doi: 10.1016/S0140-6736(14)62294-7 25706853 PMC6309957

[36] Pentz-Vidovíc I Skorić T Grubisić G Korsíc M Ivicevic-Bakulic T Besenski N. Evolution of clinical symptoms in a young woman with a recurrent gonadotroph adenoma causing ovarian hyperstimulation. Eur J Endocrinol. (2000) 143:607−14. doi: 10.1530/eje.0.1430607 11078984

[37] Shimon I Rubinek T Bar-Hava I Nass D Hadani M Amsterdam A. Ovarian hyperstimulation without elevated serum estradiol associated with pure follicle-stimulating hormone-secreting pituitary adenoma. J Clin Endocrinol Metab. (2001) 86:3635−40. doi: 10.1210/jcem.86.8.7766 11502789

[38] Schoot DC Coelingh Bennink HJ Mannaerts BM Lamberts SW Bouchard P Fauser BC. Human recombinant follicle-stimulating hormone induces growth of preovulatory follicles without concomitant increase in androgen and estrogen biosynthesis in a woman with isolated gonadotropin deficiency. J Clin Endocrinol Metab. (1992) 74:1471−3. doi: 10.1210/jcem.74.6.1592896 1592896

[39] Papanikolaou N Millar O Coulden A Parker N Sit L Kelly C. Clinical characteristics of functioning gonadotroph adenoma in women presenting with ovarian hyperstimulation: Audit of UK pituitary centres. Clin Endocrinol. (2023) 99:386−95. doi: 10.1111/cen.14949 37430451

[40] Sicilia V Earle J Mezitis SGE. Multiple ovarian cysts and oligomenorrhea as the initial manifestations of A gonadotropin-secreting pituitary macroadenoma. Endocr Pract. (2006) 12:417−21. doi: 10.4158/EP.12.4.417 16901798

[41] Christin-Maitre S Rongières-Bertrand C Kottler ML Lahlou N Frydman R Touraine P. A spontaneous and severe hyperstimulation of the ovaries revealing a gonadotroph adenoma. J Clin Endocrinol Metab. (1998) 83:3450−3. doi: 10.1210/jcem.83.10.5182 9768644

[42] Knoepfelmacher M Danilovic DLS Rosa Nasser RHR Mendonça BB. Effectiveness of treating ovarian hyperstimulation syndrome with cabergoline in two patients with gonadotropin-producing pituitary adenomas. Fertil Steril. (2006) 86:719.e15–18. doi: 10.1016/j.fertnstert.2006.01.055 16952513

[43] Pfeifer S Butts S Dumesic D Fossum G Gracia C La Barbera A. Prevention and treatment of moderate and severe ovarian hyperstimulation syndrome: a guideline. Fertil Steril. (2016) 106:1634−47. doi: 10.1016/j.fertnstert.2016.08.048 27678032

[44] Boothroyd C Karia S Andreadis N Rombauts L Johnson N Chapman M. Consensus statement on prevention and detection of ovarian hyperstimulation syndrome. Aust NZ J Obst Gynaeco. (2015) 55:523−34. doi: 10.1111/ajo.2015.55.issue-6 26597569

[45] Karapanou O Tzanela M Tamouridis N Tsagarakis S. Gonadotroph pituitary macroadenoma inducing ovarian hyperstimulation syndrome: successful response to octreotide therapy. Hormones (Athens). (2012) 11:199−202. doi: 10.14310/horm.2002.1347 22801566

[46] Castelbaum AJ Bigdeli H Post KD Freedman MF Snyder PJ. Exacerbation of ovarian hyperstimulation by leuprolide reveals a gonadotroph adenoma. Fertil Steril. (2002) 78:1311−3. doi: 10.1016/S0015-0282(02)04342-X 12477530

[47] Murakami T Higashitsuji H Yoshinaga K Terada Y Ito K Ikeda H. Management of ovarian hyperstimulation due to follicle-stimulating hormone-secreting gonadotroph adenoma. BJOG. (2004) 111:1297−300. doi: 10.1111/j.1471-0528.2004.00409.x 15521879

[48] Garmes HM Grassiotto OR Fernandes YB Queiroz L de S Vassalo J de Oliveira DM. A pituitary adenoma secreting follicle-stimulating hormone with ovarian hyperstimulation: treatment using a gonadotropin-releasing hormone antagonist. Fertil Steril. (2012) 97:231−4. doi: 10.1016/j.fertnstert.2011.10.015 22118994

[49] Winkler N Bukulmez O Hardy DB Carr BR. Gonadotropin releasing hormone antagonists suppress aromatase and anti-Müllerian hormone expression in human granulosa cells. Fertil Steril. (2010) 94:1832−9. doi: 10.1016/j.fertnstert.2009.09.032 19896656

[50] Macchia E Simoncini T Raffaelli V Lombardi M Iannelli A Martino E. A functioning FSH-secreting pituitary macroadenoma causing an ovarian hyperstimulation syndrome with multiple cysts resected and relapsed after leuprolide in a reproductive-aged woman. Gynecol Endocrinol. (2012) 28:56−9. doi: 10.3109/09513590.2011.588758 21770827

[51] De Croos M Vender J Elshimy G Stachura M. A follicular stimulating hormone secreting adenoma. J Endocr Soc. (2021) 5:A565. doi: 10.1210/jendso/bvab048.1151

[52] Roberts JE Spandorfer S Fasouliotis SJ Lin K Rosenwaks Z. Spontaneous ovarian hyperstimulation caused by a follicle-stimulating hormone-secreting pituitary adenoma. Fertil Steril. (2005) 83:208−10. doi: 10.1016/j.fertnstert.2004.06.061 15652911

